# The Psychosocial and Contextual Predictors of Revenge Desire and Attitudes in Crime Victims: A Scoping Review

**DOI:** 10.1177/00332941241313032

**Published:** 2025-01-18

**Authors:** Katie McGaughey, Rian Delaney, Emily McGlinchey, Donncha Hanna, Cherie Armour

**Affiliations:** 1596 Queen’s University Belfast, Belfast, UK; 1596 Queen’s University Belfast, Belfast, UK; The University of Ulster, Coleraine, UK; 1596 Queen’s University Belfast, Belfast, UK

**Keywords:** Predictors of revenge, revenge predictors, revenge desire, revenge attitudes, vengefulness, vengeance, crime victims, victims of crime, victimization, violence, violent crime, mental health, justice

## Abstract

Criminal victimization is associated with an increased risk of violent offending, which can be motivated by revenge. Experiencing revenge desire could also be harmful for crime victims’ mental health. To limit revenge’s harmful effects, researchers have examined the predictors of revenge desire and attitudes. However, little is known about the predictors of revenge desire and attitudes in crime victims specifically. This scoping review aims to identify the contextual and psychosocial predictors of revenge desire and attitudes from the existing literature. Databases (PsycInfo, PsycArticles, SCOPUS, Web of Science and MEDLINE) were searched in February 2024. Papers published in English, with data pertaining to the psychosocial and contextual predictors of revenge desire and attitudes in crime victims, were included. Quantitative studies and meta-analyses were included. Qualitative studies and reviews were excluded. Risk of bias was assessed using JBI’s critical appraisal tools. 3689 records were screened. 10 reports, covering 14 studies, were included. A narrative review was conducted. Four categories of predictors were identified, namely 1) offence-related predictors, 2) mental health predictors, 3) criminal justice system predictors, and 4) demographic predictors. Significant predictors included PTSD symptoms, which were correlated with higher revenge desire, and participation in restorative justice, which was found to lower crime victims’ desire for violent revenge in a series of randomized controlled trials. However, this review highlights the need for further research in this area. Many predictors were merely examined in a single study, so require replication. In addition, studies were largely cross-sectional, limiting conclusions about causation.

## Introduction

An act of revenge can be defined as a retaliatory response to a perceived harm or injustice (Stuckless & Goranson, 1994). Revenge, or the desire for it, can be triggered by interpersonal transgressions. For example, being the victim of a crime (Kunst, 2011). This review seeks to understand the predictors of revenge desire and attitudes in victims of crime. This is because revenge desire can have harmful consequences for victims of crime. For example, it has been linked to poor mental health outcomes (Stuckless, 1997). Becoming a victim of crime is also linked to an increased risk of the victim going on to offend in the future (Heber, 2014). A portion of this offending may be motivated by revenge (Heber, 2014; New York Police Department, 2012). This could involve revenge against the original perpetrator, or “displaced revenge”, which targets a third party who was not involved in the original transgression (Jackson et al., 2019). Identifying the contributing factors toward revenge desire and attitudes in victims of crime could help to limit revenge’s harmful consequences (Jackson et al., 2019).

### Defining revenge

This review focuses on the psychological experience of revenge. It is important to clarify this at the outset. This is because revenge research is an emerging area, in which conceptual and definitional disagreements abound (Boon & Yoshimura, 2020). The current paper treats revenge acts and the psychological experience of revenge as related, but separate, concepts. This review paper focuses specifically on revenge as a psychological experience. This includes both the desire for revenge, or revenge feelings, and individuals’ attitudes toward revenge. Revenge desire refers to an individual’s desire for revenge in response to a specific situation or wrongdoing (van Denderen et al., 2014). Revenge attitudes, on the other hand, refer to an individual’s level of support for revenge-taking in general (van Denderen et al., 2014). The predictors of acts of revenge, or revenge behavior, fall outside the scope of this paper.

Treating revenge desire and behavior as distinct concepts is important. This is because not everyone who experiences revenge desire intends to take revenge (Orth, 2004). Research suggests that most individuals who desire revenge do not go on to take revenge (Crombag et al., 2003; DiBlasi et al., 2024; DiBlasi & Kassinove, 2022; Lopes Cardozo et al., 2000). For example, in Crombag et al.'s (2003) study of students, 64% of participants reported experiencing revenge desire. Nineteen percent reported acting on this, and a mere 1.6% reported engaging in physical violence. Therefore, only a small minority of students who experienced a desire for revenge took violent revenge. A more recent study by DiBlasi and Kassinove (2022) found similar results, with 97% of participants experiencing thoughts of revenge, and only 19% reporting that they had acted on these thoughts. It appears that most individuals who desire revenge do not go on to take it.

The desire for revenge should not be stigmatized, as Gollwitzer (2009) argues. Desiring revenge does not equate to condoning violent or illegal acts (Orth, 2004). Instead, revenge feelings appear to be a common experience after being wronged. In qualitative studies, many victims of crime have reported experiencing a desire for revenge in the wake of the crime (Field, 2012; Stuckless, 1997). Revenge desire may even act as a coping mechanism post-crime (Orth, 2004). For example, researchers have posited that imagining oneself taking revenge post-crime could empower victims and restore their positive self-image, which is often damaged by the crime (Orth, 2004). Revenge desire therefore appears to be a common response to crime, and should not be stigmatized (Gollwitzer, 2009).

### Predictors of revenge

Previous research has focused on the predictors of revenge desire in the general population (Jackson et al., 2019). This is because revenge acts can have harmful consequences. Such consequences include violence, and even loss of life. Understanding the psychosocial and contextual predictors of revenge could help to identify those who are at risk of violent offending, reducing instances of revenge and preventing violence (Boon & Yoshimura, 2020; Field, 2012). This is important, as whilst it seems that experiencing revenge desire is common and that most of those who desire revenge do not engage in violence (Crombag et al., 2003; DiBlasi et al., 2024; DiBlasi & Kassinove, 2022), revenge desire’s contribution to violence is still significant. Jackson et al. (2019) reference statistics that demonstrate the scale of the problem. In New York, 42% of homicides were found to be motivated by revenge in 2012, and 61% of school shootings across the United States of America are thought to be motivated by revenge (New York Police Department, 2012; Vossekuil et al., 2004). Revenge can motivate violence, so understanding the predictors of revenge desire could help to prevent violence.

Previous research has explored a multitude of demographic and psychological predictors of revenge. These include the role of gender, age, emotions and personality factors, among others. The influence of gender on revenge is unclear. Some studies have found that men experience stronger revenge desire than women, whilst others have found no effect (Goldner et al., 2019). Older age has been linked to less desire for revenge (Cota-McKinley et al., 2001). This may be because older people are less impulsive, and are more likely to avoid taking revenge as they foresee its potentially negative consequences (Cota-McKinley et al., 2001). The role of the “Big 5” personality traits has also been examined, with low agreeableness and high neuroticism found to be predictive of stronger revenge desire (McCullough et al., 2001). Emotional triggers for revenge have also been explored. Perhaps unsurprisingly, anger has been found to precede revenge (DiBlasi et al., 2024; Roseman et al., 1994). Yet shame may also provoke revenge, particularly in collectivist cultures (Bloom, 2001; Shteynberg, 2005). Predictors related to the circumstances of the wrongdoing itself have also been examined. For example, when someone is wronged, revenge desire has been found to be stronger where the wrongdoing is seen as deliberate (Ames & Fiske, 2013; Gray & Wegner, 2009). Broader contextual factors around the wrongdoing may also influence revenge predictors. For example, revenge is more likely to happen in workplaces where employees do not feel supported and where there is low procedural justice (Aquino et al., 2006). Since contextual factors influence revenge, researchers have examined revenge predictors in specific contexts. This includes the workplace, as previously mentioned, and also romantic relationships (Boon et al., 2009; Tripp & Bies, 2010). However, one context in which the predictors of revenge are not well-understood is in victims of crime (Orth, 2004).

### Predictors of revenge in victims of crime

There are many potential predictors of revenge which are specific to the context of criminal victimization (Orth, 2003). Victims’ experiences within the criminal justice system may impact their revenge desire (Orth, 2004). For example, if the perpetrator is identified and sentenced, then victims’ revenge desire may be lower than it would be if the offender were still at large (van Denderen et al., 2014). This is because the victim may feel that justice has been done, so they do not need to take the pursuit of justice into their own hands (Gerber & Jackson, 2013; Osgood, 2017). The type of crime a victim experiences may also impact their revenge desire. For example, violent crime could lead to stronger revenge desire than property crime. Violent assault could be perceived by the victim as a personal attack, rather than an opportunistic crime (Hannon, 2002). This could mean that violent assaults damage victims’ self-image than property crimes, increasing victims’ revenge desire in order to bolster their self-image (Gerlsma & Lugtmeyer, 2018; Orth, 2004). However, this is poorly understood (Carmody & Gordon, 2011). And despite the many potential predictors of revenge which are specific to crime victims, existing literature on the predictors of revenge desire in victims of crime is limited and disparate.

### Importance of researching revenge in crime victims

Increasing understanding of revenge desire and attitudes in victims of crime is important for several reasons. Firstly, as noted above, violence prevention is an important driver of research on revenge in the general population (Jackson et al., 2019). Criminal victimization is highly relevant here. This is because experiencing criminal victimization is linked to an increased risk of victims later committing violent crime themselves (Heber, 2014). This may be motivated by revenge (New York Police Department, 2012). Therefore, victims of crime are a particularly important population in which to study revenge desire for the purposes of violence prevention. Knowing the predictors of revenge desire could help us to identify those victims of crime who are at risk of offending and intervene to stop it (Gerlsma & Lugtmeyer, 2018). It could also inform practical interventions, which could reduce instances of revenge. One such intervention could involve adapting addiction-treatment techniques for violence prevention. Chester and DeWall (2017) posit that addiction treatments could help to reduce retaliatory aggression, based on their finding that revenge activates similar reward-processing centers of the brain to those which are implicated in narcotics addiction (i.e., the striatum and nucleus accumbens). Moreover, community activity-based interventions are also promising. One study found that adolescent victims of violent crime experienced lower revenge desire, and were less likely to engage in firearm aggression, after taking part in a community activity-based intervention (Lee et al., 2022). This illustrates the potential for interventions with victims of crime to lower revenge desire and prevent real-world instances of violence. Such interventions could be improved upon and informed by better understanding of the predictors of revenge desire in victims of crime (Lee et al., 2022).

Additionally, victims of crime often experience mental health problems in the wake of the crime (Kilpatrick & Acierno, 2003). These problems can persist long after the criminal justice process is complete (Parsons & Bergin, 2010). Revenge desire could play a key role in this, as experiencing long-term revenge desire after being wronged is linked to negative affect, depression and anxiety, as well as lower quality of life (Barcaccia et al., 2022; Stuckless, 1997). Several studies have also linked revenge desire to PTSD in victims of crime (Horowitz, 2007; Kunst, 2011). Yet, whilst a link between revenge desire and poor mental health has been established, the exact nature of this relationship, and its predictors, is not well understood (Kunst, 2011). Understanding the predictors of revenge desire in victims of crime could improve support for victims’ mental health. This information could inform both clinicians and support services (Field, 2012).

### The current review

Research on the predictors of revenge desire among victims of crime is an emerging area. Current quantitative research on this topic is limited and disparate. Yet, as demonstrated above, it could play an important role in informing violence prevention strategies and improving mental health support for crime victims (Horowitz, 2007; Lee et al., 2022). There is a need to collate existing research, and provide an overview of revenge predictors in victims of crime. Informative literature reviews on the predictors of revenge have been carried out when it comes to the general population, with one meta-analysis specifically examining narcissism as a predictor of vengeance (Jackson et al., 2019; Rasmussen, 2016). However, there are no existing reviews of the literature on the predictors of revenge desire in crime victims. The current review therefore aims to identify the predictors of revenge desire and attitudes in crime victims from existing literature. Given the lack of research in this area and its emerging nature (Orth, 2004), the current review has a broad focus. It aims to provide a comprehensive overview of the general predictors of revenge desire and attitudes in victims of crime, examining both contextual and psychosocial predictors.

## Method

### Design

A scoping review was conducted, with the aim of identifying the predictors of revenge desire and attitudes in victims of crime. A scoping review was chosen because the aim of this paper is to scope the literature on the predictors of revenge desire and attitudes in victims of crime, and to identify knowledge gaps (Munn et al., 2018).

### Search strategy

The following databases were searched: PsycInfo, PsycArticles, SCOPUS, Web of Science and MEDLINE. Searches were conducted in February 2024. Boolean operators were used. See Appendix A for search details. The following terms were entered in all databases (with minor adjustments according to the parameters of each database e.g., quotation marks around phrases were not required for PsycInfo):

(revenge OR vengeance OR vengefulness OR retaliat*) AND victim* AND (homicide OR murder OR manslaughter OR infanticide OR filicide OR “dangerous driving” OR “inconsiderate driving” OR injury OR assault OR harassment OR “hate crime” OR stalk* OR “sexual assault” OR “sex* offence” OR “sexual abuse” OR rape OR groom* OR exposure OR voyeur* OR “violent crime” OR violence OR burglary OR robbery OR theft OR fraud OR blackmail OR vandal* OR arson OR firearm* OR weapon* OR “criminal damage” OR “child abuse” OR cybercrime OR “domestic violence” OR terror* OR “physical abuse” OR kidnap* OR incest OR traffick* OR “antisocial behavio*” OR “criminal behavio*” OR crime)

In selecting terms for the list of crimes, a comprehensive table of offences published by the United Kingdom’s Crown Prosecution Service was used as a starting point. A link can be found here: https://www.cps.gov.uk/sites/default/files/documents/legal_guidance/annex-1b-table-of-offences-scheme-c-class-order.pdf. All interpersonal crimes, which could be considered to have a person or group of persons as a perpetrator, and an individual person as a victim, were included. Keywords for these crimes were identified. References from included papers were considered. Duplicate papers were identified by the electronic reference manager Rayyan (Ouzzani et al., 2016), before being manually checked and removed.

### Study selection criteria

Titles and abstracts were manually reviewed by the lead author to identify relevant papers. 10% of these papers were randomly selected to be assessed by a second reviewer. A full text review was then conducted. All papers selected for full text review were considered by both the lead author and second reviewer. Papers were considered in relation to the following inclusion criteria:

Inclusion criteria: (a) peer-reviewed study (grey literature was excluded), (b) published in English, (c) includes study results pertaining to the predictors of revenge desire, (d) crime victims are included in the population and examined separately from non-crime victims in at least one statistical analysis, (e) includes at least one measure of revenge desire or attitudes, and (f) a quantitative study or a meta-analysis.

This left a total of 10 reports, containing 14 studies, for inclusion. A PRISMA flow chart was created to summarize the process. This can be found below (Figure 1).

**Figure 1.**
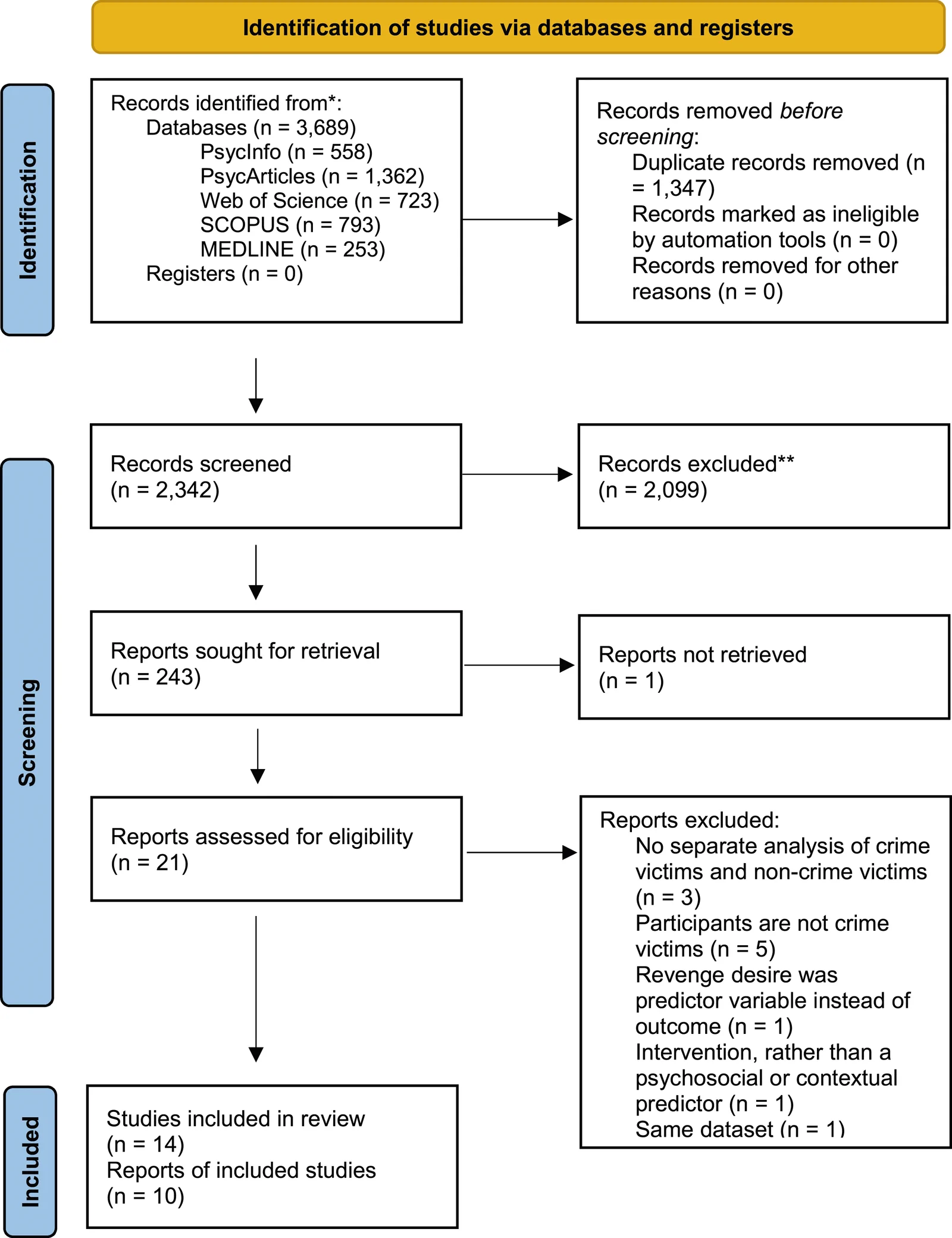
Prisma flow chart.

### Data extraction

The following data items were extracted from each included paper: title, author/s, year of publication, journal and publisher, country in which the study took place, participants’ nationality, research aims, study design, study method, data analysis method, psychosocial and contextual predictors included, revenge measure/s used, measure/s used for the psychosocial and contextual predictors, age range (in years), mean participant age, standard deviation of participants’ age, participants’ gender, crime type, results, sample size, funding source/s. See Table 1, Table 2, Table 3, and Table 4 for a summary of these findings.

**Table 1.** Sample Characteristics.

Study	*N*	Country	Crime Type	Mean Age (SD)	% of Females
1. Copeland-Linder et al. (2007)	336	United States	Physical assault	12.89 (1.5)	32
2. Davidson et al. (2013)	503	United States	Sexual violence	19.89 (2.09)	100
3. Franczyk-Glita et al. (2018)	60	Poland	Intimate partner violence	Not specified	100
4. Gerlsma & Lugtmeyer (2016)	379	Netherlands	Minor offenses, property crime, physical assault, sexual violence, Fire-setting	18.08 (1.81)	56.9
5. Kunst (2011)	207	Netherlands	Violent assault	45 (16.3)	62.8
6. Lenferink et al. (2022)	209	Netherlands	Road traffic accident bereavement	51.96 (13.09)	73.2
7a. Orth (2004)	174	Germany	Physical assault and sexual violence	37.5 (12.2)	84
7b. Orth (2004)	31	Germany	Physical assault and sexual violence	36.6 (12.8)	80.6
8. Orth et al. (2006)	174	Germany	Physical assault and sexual violence	37.5 (12.2)	84
9a. Sherman et al. (2005)	81	Australia	Violent assault	28	27
9b. Sherman et al. (2005)	151	Australia	Property crime	25	48
9c. Sherman et al. (2005)	69	United Kingdom	Robbery	36	44
9d. Sherman et al. (2005)	145	United Kingdom	Burglary	31	49
10. van Denderen et al. (2014)	331	Netherlands	Homicidal bereavement	52.6 (15.5)	65.9

**Table 2.** Measures of Revenge and Predictors Included in Analyses.

Study	Revenge Measure	Predictors Included	Predictor Measures
1. Copeland-Linder et al. (2007)	Children’s Perceptions of Environmental Violence (Retaliation Subscale) (Hill & Noblin, 1991)	SES, age, parents' attitudes towards fighting and youth perceptions of parents' attitudes towards fighting, past exposure to violence, anxiety/depression, aggression and aggressive peers, gender, relationship with the perpetrator, intentionality of crime	Parents’ attitudes toward fighting and youth perceptions of parents' attitudes towards fighting (P. Orpinas et al., 1999); exposure to violence (Richters & Martinez, 1993); anxiety/depression measured using the Child Behavior Checklist (Achenbach, 1991); aggressive peers (Simons-Morton et al., 1999); aggression (Orpinas, 1993); additional predictors measured using authors' own demographic questionnaires
2. Davidson et al. (2013)	Transgression-related Interpersonal Motivations Scale (TRIM-18) (McCullough et al., 1998)	Sexual violence experiences	Sexual assault experiences measured using the Sexual Experiences Survey—short form victimization (SES-SFV) (Koss et al., 2007)
3. Franczyk-Glita et al. (2018)	Psychological Inventory of Aggression Syndrome (Gaś, 1988)	Intimate partner violence experiences, age, education, length of relationship, length of violence, partner’s age, partner’s education	Predictors measured using authors' own demographic questionnaires
4. Gerlsma & Lugtmeyer (2016)	Transgression-related Interpersonal Motivations Scale (TRIM-18) (McCullough et al., 1998)	Type of crime, crime victim status, gender, age, time since incident	Predictors measured using authors' own demographic questionnaires
5. Kunst (2011)	3-item revenge scale for use with crime victims (Orth, 2003)	PTSD, perpetrator punishment severity, age, gender, time since victimization, level of compensation for pain and suffering, sexual violence, physical violence (severe or moderate), theft with violence	Dutch version of PTSD Symptom Scale, Self-Report Version (PSS-SR) (Arntz, 1993; Foa et al., 1993)
6. Lenferink et al. (2022)	Dutch version of Posttraumatic Anger Questionnaire (PAQ) (Orth & Maercker, 2009)	PTSD, prolonged grief disorder	20-Item PTSD Checklist for DSM-5 (PCL-5) (Blevins et al., 2015)
7a. Orth (2004)	3-item revenge scale for use with crime victims (Orth, 2003)	Perpetrator punishment severity, compensation, acknowledgement of wrongdoing. Controlled for: age, gender, assault type, time since victimization, and victim-perpetrator relationship	Predictors measured using authors' own demographic questionnaires
7b. Orth (2004)	3-item revenge scale for use with crime victims (Orth, 2003)	Time (pre-trial at T1 vs. post-trial at T2), perpetrator punishment severity, compensation, acknowledgement of wrongdoing	Predictors measured using authors' own demographic questionnaires
8. Orth et al. (2006)	3-item revenge scale for use with crime victims (Orth, 2003)	PTSD	PTSD measured using the Impact of Event scale—Revised (IES-R) (Maercker & Schützwohl, 1998; Weiss & Marmar, 1997)
9a. Sherman et al. (2005)	Single item: “Would you do some harm to the offender yourself if you had the chance?”	Restorative justice conference participation	Diversion from prosecution to a restorative justice meeting, compared to control group going through criminal justice prosecution
9b. Sherman et al. (2005)	Single item: “Would you do some harm to the offender yourself if you had the chance?”	Restorative justice conference participation	Diversion from prosecution to a restorative justice meeting, compared to control group going through criminal justice prosecution
9c. Sherman et al. (2005)	Single item: “Do you wish you could physically retaliate against the offendernow?”	Restorative justice conference participation	Diversion from prosecution to a restorative justice meeting, compared to control group going through criminal justice prosecution
9d. Sherman et al. (2005)	Single item: “Do you wish you could physically retaliate against the offendernow?”	Restorative justice conference participation	Diversion from prosecution to a restorative justice meeting, compared to control group going through criminal justice prosecution
10. van Denderen et al. (2014)	Transgression-Related Interpersonal Motivations Scale (TRIM-18) (McCullough et al., 1998); vengeance scale (Stuckless & Goranson, 1994)	PTSD, complicated grief, positive functioning, sex, juridical status, relationship with perpetrator, time since loss, age	Dutch version of PTSD Symptom Scale, Self-report Version (PSS-SR) (Arntz, 1993; Foa et al., 1993); Dutch version of the Inventory of Complicated Grief (ICG) (Prigerson et al., 1995); positive functioning measured using the Positive Outcome Scale (POS) (Appelo, 2005; Appelo & Harkema-Schouten, 2003); other predictors measured using authors' own demographic questionnaires

**Table 3.** Information on Type of Analysis Used, Predictors Included and the Significant Predictors.

Study	Analysis Used	Predictors Examined	Significant Predictors	Non-significant predictors
1. Copeland-Linder et al. (2007)	Correlations, hierarchical regression	SES, age, parents' attitudes towards fighting and youth of parents' attitudes towards fighting, gender, relationship with the perpetrator, past exposure to violence, depression/anxiety, intentionality, aggression and aggressive peers	Significant predictors of higher revenge desire in the regression model were: lower socioeconomic status, older age, more affiliating with aggressive peers (only significant for boys, not girls), and higher adolescent perceptions that their parents supported fighting. Higher aggression was significant only in girls.	Non-significant predictors were: gender, anxiety, depression, aggression (not significant in overall sample), victims' perception of whether the offence was intentional.
2. Davidson et al. (2013)	Correlations, multivariate regression	Sexual violence experiences	Experiencing more instances of sexual violence was significantly correlated with experiencing higher revenge desire, and a significant predictor of revenge desire in the regression model.	n/a
3. Franczyk-Glita et al. (2018)	Mann-Whitney U tests, correlations	Intimate partner violence experiences, age, education, length of relationship, length of violence, partner’s age, partner’s education	n/a	Non-significant predictors were: intimate partner violence experiences, age, education, length of relationship, length of violence, partner’s age, partner’s education.
4. Gerlsma & Lugtmeyer (2016)	Correlations, linear regression	Type of crime, crime victim status, gender, age, time since incident	Significant predictors of revenge desire in the regression model were: gender, with men experiencing more revenge desire than women; crime type, with sexual violence survivors being significantly less likely to desire revenge than victims of property and physical assault; crime victim status, as people who were victims of crime were significantly more likely to experience revenge desire than non-crime victims. Correlation analyses indicated that the following predictors were significant: time since victimization, as where less time had passed since victimization, revenge feelings were significantly higher; age, with younger age associated with higher revenge desire.	n/a
5. Kunst (2011)	Regression	PTSD, perpetrator punishment severity, age, gender, time since victimization, level of compensation for pain and suffering, sexual violence, physical violence (severe or moderate), theft with violence	Experiencing the re-experiencing/intrusion symptom cluster of PTSD was a significant predictor of higher revenge desire. Higher perceived offender punishment severity was significantly correlated with lower revenge desire (but non-significant after adjusting for PTSD symptoms).	Non-significant predictors were: age, gender, time since victimization, level of compensation for pain and suffering, sexual violence, physical violence (severe or moderate), theft with violence, PTSD avoidance and hyperarousal clusters.
6. Lenferink et al. (2022)	Confirmatory factor analysis	PTSD, prolonged grief disorder	Confirmatory factor analyses indicated that higher desire for revenge was significantly correlated with higher PTSD scores.	Prolonged grief disorder was not significantly correlated with revenge desire.
7a. Orth (2004)	Correlations, hierarchical regression	Perpetrator punishment severity, compensation, acknowledgement of wrongdoing. Controlled for: age, gender, assault type, time since victimization, and victim-perpetrator relationship.	Regression analysis indicated that compensation was a significant predictor of current revenge feelings, with higher satisfaction with compensation linked to lower revenge desire. In correlation analyses, initial feelings of revenge after the crime and current feelings of revenge were assessed separately. For initial feelings, significantly correlated predictors were: punishment severity (higher punishment severity linked to lower revenge), compensation (higher compensation linked to lower revenge), acknowledgement of wrongdoing (linked to lower revenge), age (higher age linked to lower revenge), gender (males experienced more revenge feelings), and assault type, with non-sexual violent assault linked to higher revenge feelings. For current revenge feelings, correlation analyses indicated that punishment severity (higher punishment severity linked to lower revenge), compensation (higher compensation linked to lower revenge), acknowledgement of wrongdoing (linked to lower revenge) and age (higher age linked to lower revenge) were significantly linked to revenge feelings.	In regression analyses for current revenge feelings, the following predictors were not significant: age, gender, assault type, victim-perpetrator relationship, time since victimization, acknowledgement of wrongdoing, punishment severity. In correlation analyses for initial feelings of revenge after the crime, victim-perpetrator relationship and time since victimization were not significant. For current revenge feelings, correlation analyses indicated that gender, assault type, victim-perpetrator relationship and time since victimization were not significant.
7b. Orth (2004)	Correlations, hierarchical regression	Perpetrator punishment severity, compensation, acknowledgement of wrongdoing	Correlation analyses indicated that punishment severity was associated with a change in feelings of revenge between T1 (before criminal trial) and T2 (after criminal trial), with greater punishment severity being associated with reduced revenge feelings.	Compensation and acknowledgement of wrongdoing were not significantly associated with a change in revenge feelings between T1 and T2.
8. Orth et al. (2006)	Correlations, hierarchical regression, moderation, mediation	PTSD, physical harm, emotional support, age, time since victimization, retaliation motive	Feelings of revenge were significantly correlated with the intrusion and hyperarousal symptom clusters of PTSD, with more intrusion and hyperarousal symptoms relating to more revenge feelings. Revenge feelings were also significantly correlated with age (lower age was related to higher revenge feelings), physical harm and retaliation motive. Time since victimization moderated the relationship between revenge feelings and hyperarousal, but not with avoidance.	The avoidance symptom cluster of PTSD was not significantly correlated with revenge. Emotional support and time since victimization were not significantly correlated with revenge.
9a. Sherman et al. (2005)	Meta-analysis	Restorative justice conference participation	Feelings of revenge were significantly lower in the restorative justice group compared to the control group, who had experienced traditional criminal justice processes.	n/a
9b. Sherman et al. (2005)	Meta-analysis	Restorative justice conference participation	Feelings of revenge were significantly lower in the restorative justice group compared to the control group, who had experienced traditional criminal justice processes.	n/a
9c. Sherman et al. (2005)	Meta-analysis	Restorative justice conference participation	Feelings of revenge were significantly lower in the restorative justice group compared to the control group, who had experienced traditional criminal justice processes.	n/a
9d. Sherman et al. (2005)	Meta-analysis	Restorative justice conference participation	Feelings of revenge were significantly lower in the restorative justice group compared to the control group, who had experienced traditional criminal justice processes.	n/a
10. van Denderen et al. (2014)	Correlations, linear regression, t-test, ANOVA	PTSD, complicated grief, positive functioning, sex, juridical status, relationship with perpetrator, time since loss, age, homicidal bereavement experiences	Correlation analyses indicated that PTSD and complicated grief were significantly associated with revenge desire and attitudes, with higher PTSD and complicated grief symptoms linked to greater revenge, and higher positive functioning was significantly associated with lower revenge desire and attitudes. Age was significantly correlated with revenge feelings, with higher age associated with lower revenge feelings. ANOVA results revealed that the victim-perpetrator relationship was a significant predictor of revenge desire (but not revenge attitudes), with lower revenge desire where the perpetrator was a direct family member, rather than acquaintance, friend, stranger or indirect family member. T-tests revealed that experiences of homicidal bereavement were significantly associated with higher revenge feelings, compared to a sample of students who had experienced more minor interpersonal transgressions.	Gender and the juridical status of the perpetrator were not significant predictors of revenge feelings or attitudes. Victim-perpetrator relationship was not a significant predictor of revenge attitudes. Age was not correlated with revenge attitudes.

**Table 4.** Master List of Predictors.

Contextual Predictors
Offence-Related Predictors
Type of crime**
Victimization
Crime victim status (whether an individual had experienced crime or not)**
Sexual violence experiences**^1^
Intimate partner violence experiences^1^
Physical violence (severe or moderate)^1^
Theft with violence^1^
Nature of victim-perpetrator relationship*
Perceived intentionality of crime^1^
Time since crime*
Multiple instances of victimization**^1^
Perpetrator acknowledgement of wrongdoing*^1^
Criminal justice system-related predictors
Perpetrator punishment severity*
Time (pre-trial vs. post-trial)**^1^
Level of compensation for pain and suffering*
Restorative justice conference participation**
Juridical status^1^
Psychosocial predictors
Mental health-related predictors
PTSD**
Prolonged grief disorder*
Anxiety^1^
Depression^1^
Positive functioning**^1^
Demographic predictors
Gender*
Age*
Socioeconomic status**^1^
Education^1^
Parents' attitudes towards fighting^1^
Aggressive peers^1^
Youth perceptions of parents' attitudes towards fighting**^1^
Past exposure to violence^1^
Partner’s age (in context of intimate partner violence)^1^
Partner’s education (in context of intimate partner violence)^1^
Miscellaneous predictors
Aggression^1^

*Note*. * = predictor’s significance is inconclusive due to mixed results. ** = significant predictor. ^1^ = predictor examined in a single study.

### Quality appraisal/risk of bias

Risk of bias was assessed using JBI’s critical appraisal tools (Barker et al., 2023; Moola et al., 2020). All included studies were of fair to high quality, scoring at least 62.5% (*M* = 82.44%). Scores ranged from 62.5%–100%. A summary of scores can be found below, in Figure 2. The most common problem observed in the cross-sectional studies was a failure to identify confounding factors (1,2). One paper provided limited detail in its description of participants (4). Another problem was observed in paper 9, a meta-analysis of four randomized controlled trials (9a,9b,9c,9d). This paper examined the influence of restorative justice conferences on crime victims’ desire for violent revenge. Victims were randomly assigned to either experience restorative justice conferences, or traditional criminal justice processes. However, individuals delivering treatment were not blind to treatment assignment.

**Figure 2.**
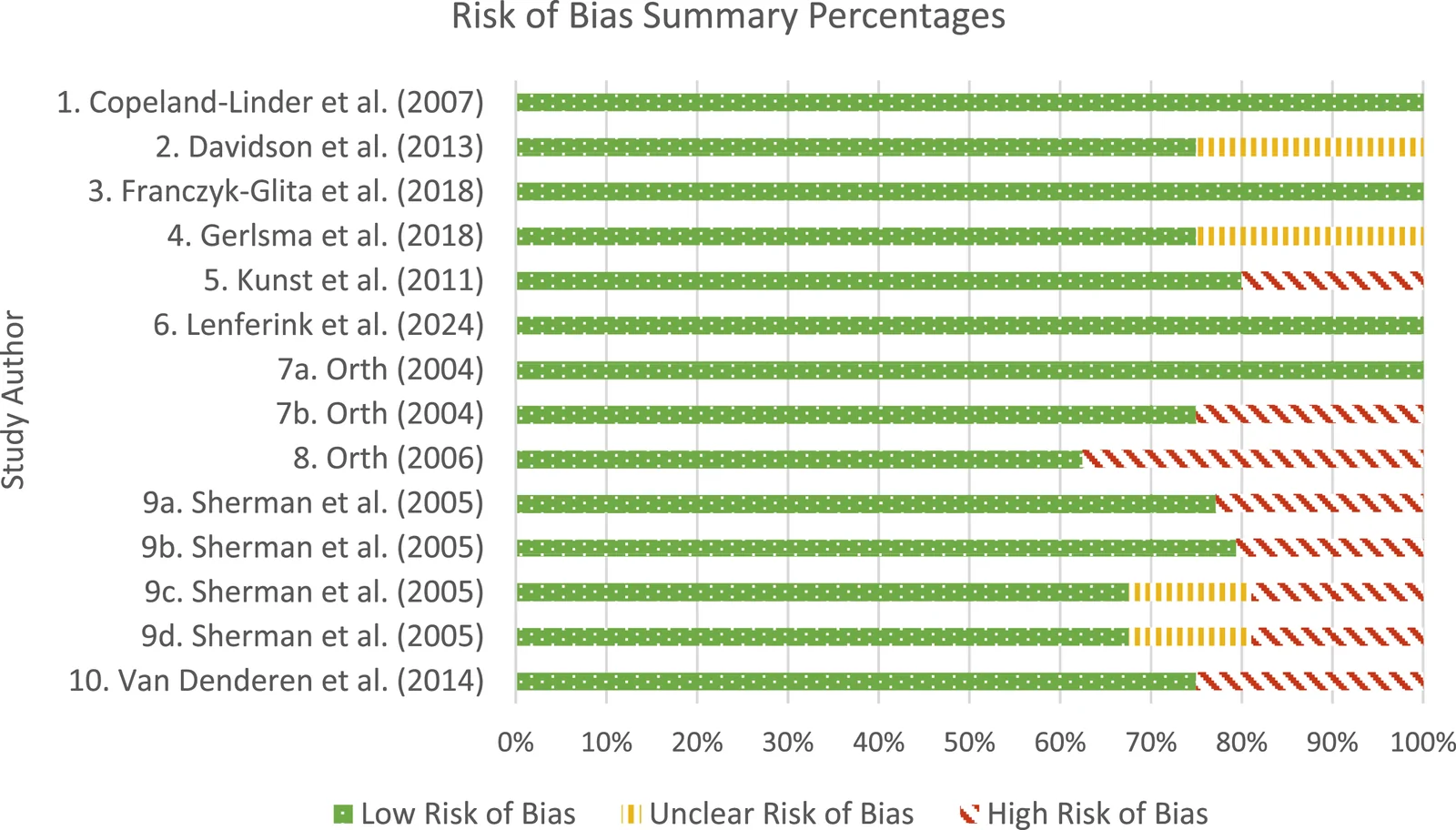
Risk of bias scores.

## Results

All studies included in the review were conducted in Western countries. The majority of studies were conducted in European countries, including Germany, the Netherlands, Poland, and the United Kingdom. Several studies were also conducted in Australia and the United States. The studies included both adolescent and adult participants, with participants’ mean ages ranging from 12.89 (1) to 52.6 (10). Two studies focused exclusively on female victims of crime (2, 3). All other studies included both male and female participants (1, 4, 5, 6, 7a, 7b, 8, 9a, 9b, 9c, 9d, 10). In terms of crime type, the majority of studies focused on violent assault, sexual violence, or property crimes (1, 2, 4, 5, 7a, 7b, 8, 9a, 9b, 9c, 9d). One study focused exclusively on survivors of intimate partner violence (3). Another focused only on victims of road traffic accidents (6), and one study exclusively examined victims of homicidal bereavement (10). Further detail on the demographics of the included studies can be found in Table 1. Narrative synthesis was carried out. Studies were grouped into four categories. These categories were guided by the research question, which sought to identify the psychosocial and contextual predictors of revenge desire and attitudes in victims of crime. Two categories of contextual predictors of revenge desire and attitudes in victims of crime were identified. These were 1) offence-related predictors, which were contextual predictors related to the circumstances of the crime itself, and 2) criminal justice system-related predictors, which included predictors related to victims’ experiences within the criminal justice system. Two categories of psychosocial predictors of revenge desire and attitudes in victims of crime were identified. These were: 1) mental health or well-being related predictors, and 2) sociodemographic predictors. Further detail on the predictors can be found in Table 2 and Table 4.

## Discussion

### Summary

This review has revealed that research on the predictors of revenge desire and attitudes among victims of crime is limited. Many of the reviewed predictors were merely examined in a single study. The need for further research in this area is evident. Nevertheless, there have been some interesting findings, which could inform future research. Four main categories of predictors were identified. Namely, offence-related predictors, or contextual predictors related to the circumstances around the crime itself, criminal justice system-related predictors, victim mental health-related predictors, and sociodemographic predictors. These predictors are explored in more detail below. Summaries of significant and non-significant predictors can be found in Table 2 and Table 4.

### Offence-related predictors

Offence-related predictors were defined as contextual predictors which relate to the circumstances of the crime itself. Seven offence-related predictors were identified (1, 2, 3, 4, 5, 7a, 7b, 10). Not all of these predictors were significant. See Table 2 for a summary. Of the seven factors, three were only examined in a single study. Predictors examined in a single study included: experiencing multiple instances of criminal victimization (2), whether the victim perceived the crime as intentional (1), and whether the offender had acknowledged their wrongdoing (7a). Further research is needed to replicate these findings. Predictors explored across multiple studies included: experiencing criminal victimization itself (note that this predictor includes multiple sub-predictors, see Table 4 above for details) (1,2,10), the victim-perpetrator relationship (1, 7a, 10), time since the crime (4, 5, 7a, 7b, 10), and crime type (4). However, results on these predictors were mixed, again pointing to a need for more research. These factors are explored in more detail below.

Three studies found that being victimized by crime was a significant predictor of revenge feelings (1,2,10). In other words, the experience of criminal victimization was a predictor in itself. This accords with previous qualitative research, in which many victims have reported experiencing revenge desire after the crime (Field, 2012; Stuckless, 1997). It underlines the need to study crime victims’ experiences of revenge specifically. However, one study contradicted the majority, and this was a finding that experiencing domestic violence was not a significant predictor of revenge desire (3). It may be that intimate partner violence elicits relatively low revenge motivation compared to other crimes. This could be because the perpetrator and survivor-victim are in an intimate relationship. Revenge desire has been found to be lower where the wrongdoer and victim are in a close relationship or dependent on one another (Finkel et al., 2002; Hruschka & Henrich, 2006).

Relatedly, three studies specifically examined whether the victims’ relationship with the perpetrator predicted their revenge feelings (1, 7a, 10). Two studies compared those who were victimized by strangers to those who were victimized by someone they knew, finding no significant relationship with revenge desire (1, 7a). This indicates that revenge desire in crime victims is not impacted by whether the offender is a stranger or an acquaintance. This contradicts research findings in the wider population, whereby closer relationships have been linked to experiencing lower revenge feelings after a transgression, potentially because victims are motivated to maintain the relationship (Finkel et al., 2002; Hruschka & Henrich, 2006). However, another study examined crime victims’ relationship with the perpetrator in greater detail and found a significant effect (10). Revenge desire was lower where the perpetrator was a direct family member, compared to a stranger, friend, indirect family member or acquaintance. This suggests that the nature of the relationship between victim and offender may impact revenge feelings, with offences perpetrated by close family members provoking less revenge desire. However, this is a tentative conclusion. Since there was only one study which examined this, it is evident that further research is needed here, exploring the nature of victim-offender relationships in greater detail.

Four papers, including five studies, examined whether time since victimization predicted crime victims’ revenge feelings (4, 5, 7a, 7b, 10). It may be expected that revenge feelings would be strongest in the immediate aftermath of the crime. Several studies have linked vengeful feelings to anger and shame, and such emotions could be most heightened at this stage (Bloom, 2001; Roseman et al., 1994; Thomaes, 2006). One study found that where less time had passed since the crime, victims’ revenge feelings were significantly stronger (4). However, five studies found no significant relationship between time since victimization and revenge desire (5, 7a, 7b, 10). Yet it is noteworthy that most of these studies were cross-sectional. Most participants were reporting their feelings several years after the crime, which may reduce the reliability of these findings. For example, these studies compared revenge feelings in victims who had been victimized three years before to those who had been victimized five years before. A relationship may have been found here if victims’ revenge feelings were captured in the immediate aftermath of the offence. Alternatively, it may be that for some victims, revenge feelings do not dissipate with the passage of time. Indeed, revenge feelings may grow with time (Lee et al., 2014). Revenge can be enacted many years after the offence (Feuer & Singer, 2017). It is evident that further research is needed here, particularly longitudinal research which tracks changes in victims’ revenge feelings over time. This could be important for violence prevention as it could help to identify when violent retaliation is most likely to happen.

Whether the offender acknowledged their wrongdoing was only explored in one paper (7a). Acknowledgement of wrongdoing did not impact the victim’s revenge desire when it was measured at a point several years after the crime. This contradicts previous research findings, in which acknowledgement of wrongdoing was found to satisfy participants’ desire for revenge. Several studies have found that a key motivator for revenge is having the wrongdoer understand their mistake and learn from it, so one would expect that an offender acknowledging their wrongdoing would lower victims’ revenge desire (Funk et al., 2014; Gollwitzer et al., 2011). Further research is therefore needed here, particularly examining how acknowledgement of wrongdoing could impact victims’ revenge desire in the short-term, given that this was only measured at a point several years after the crime. Additionally, one study (1) assessed whether revenge attitudes were stronger when the victim perceived the crime as intentional. A study of adolescents who had been physically assaulted found that viewing the crime as intentional did not predict revenge attitudes. This contradicts previous literature on revenge. Deliberate transgressions are typically judged as morally worse than accidental transgressions (Gray & Wegner, 2009). Revenge tends to be triggered by wrongdoing which is perceived to violate moral or ethical norms (Jackson et al., 2019). Transgressions which are viewed as morally worse could therefore be expected to induce greater revenge desire. Lower revenge desire has also been observed in situations where the wrongdoer is viewed as less culpable for their actions. For example, where the transgressor is a child or an animal (Ames & Fiske, 2013). Further research is clearly needed on the relationship between victims’ perceptions of wrongdoing as intentional and their revenge desire.

### Mental health predictors

Five predictors focused on the relationship between victims’ mental health and revenge desire (1,5,6,8,10,11). As above, not all of these predictors were significant. See Table 2 and Table 4 for a summary. It should be noted that the role of mental health in predicting revenge desire is unclear. This is because the direction of the relationship between victims’ mental health and revenge feelings is difficult to determine from the existing, largely cross-sectional literature. For example, one pathway could be that pre-existing symptoms of depression, or symptoms which develop in the wake of victimization, provoke more rumination about the crime, making experiencing revenge feelings more likely (Copeland-Linder et al., 2007). On the other hand, intensive revenge fantasies could mean that victims struggle to move on from the crime. This could increase negative affect and promote avoidance of painful feelings. This could have deleterious impacts on crime victims’ mental health (Orth et al., 2006). A bi-directional relationship is also possible. Further research is needed to determine the exact nature and direction of this relationship, particularly longitudinal research which examines changes in victims’ mental health and revenge feelings over time. Moreover, four of the identified mental health predictors, or the majority, were only examined in a single study. These were depression, anxiety, positive functioning, and complicated grief (1,11). The need for more research on these factors is clear. Yet one predictor, PTSD, was examined across four studies (5,6,8,10). These studies linked higher PTSD, specifically the re-intrusion symptom cluster, to stronger revenge feelings. However, the exact nature of this relationship is not well-understood.

Anxiety and depression were not significantly associated with revenge attitudes in adolescents who had been assaulted (1). This contradicts previous research in the general population, which has found a link between revenge desire, anxiety, and depression (Barcaccia et al., 2022). Further research is needed to replicate this finding (1), and to explore the potential relationship between anxiety, depression and revenge desire in victims of crime. Research on whether depression and anxiety predict revenge desire and attitudes should also go beyond adolescents and focus on adult crime victim populations. This is important because there may be age-related differences in the predictors of revenge desire (Copeland-Linder et al., 2007). For example, revenge can be motivated by a desire to protect one’s reputation, positive self-image, or identity when these are threatened by victimization. Adolescents may be particularly motivated by this, since adolescence is a time when identity is developing (Erikson, 1968). The need for more research on anxiety, depression and revenge is apparent.

Complicated grief and positive functioning were examined in a single study (11). Higher complicated grief and lower positive functioning were associated with stronger revenge feelings and attitudes in victims of homicidal bereavement. However, additional research is needed to corroborate these findings. As noted above, the direction of this relationship is difficult to determine. For example, as the authors note, it may be that revenge feelings contribute to lower positive functioning by triggering negative emotions, difficulty coping with the trauma and lower quality of life (11). At the same time, lower positive functioning could trigger negative affect and lower self-efficacy. This could cause victims to turn to revenge fantasies in an attempt to restore their self-efficacy (Orth, 2004). The relationship may even be bi-directional, with revenge desire worsening mental health, and poorer mental health, in turn, contributing to stronger revenge desire. The need for more research on complicated grief, positive functioning, and revenge, is clear.

Four studies examined the relationship between PTSD and revenge desire (5,6,8,10). Across these four studies, PTSD was correlated with stronger feelings of revenge. One of these studies found that only one PTSD symptom cluster significantly predicted revenge desire (5). This was the re-intrusion and re-experiencing cluster of PTSD symptoms (5). However, the reasons for this association are unclear. Kunst (2011) posit that the link between revenge and PTSD could be explained by revenge fantasies and PTSD-related re-intrusions sharing the same cognitive content, such as thoughts or images of vengeance (Berry et al., 2005). Alternatively, revenge and PTSD-related re-intrusions may share un underlying predictor, such as rumination. Several studies have found that rumination predicts PTSD symptoms, and rumination is also correlated with revenge feelings (McCullough et al., 2001; Michael et al., 2007) and experiences of re-intrusion (Evans et al., 2007). However, Ehlers et al. (2004) argue that rumination and re-intrusion are fundamentally different experiences. Rumination involves thinking and evaluation, whilst intrusive memories involve sensory impressions. It is evident that, whilst revenge feelings and PTSD appear to correlate in crime victims, the nature of the relationship is contested. Additional research is needed to understand the exact nature of this relationship. This could help to improve mental health support for crime victims, who frequently report experiencing PTSD symptoms (Kilpatrick & Acierno, 2003).

### Criminal justice system-related predictors

Four contextual predictors related to victims’ experiences of the criminal justice system were identified (5, 7a, 7b, 10, 11). Two of these factors were only examined in a single study, so require further research. These factors were victims’ satisfaction with the compensation they received (7b) and the juridical status of the crime at the time of the study (11). Other predictors included the victim’s perception of offender punishment severity (5, 7a, 7b) and participation in restorative justice conferences (9a, 9b, 9c, 9d). Not all predictors were significant; see Table 2 and Table 4.

Three studies, across two papers, examined the relationship between revenge feelings and offender punishment severity (5, 7a, 7b). Results were mixed. In two of the three studies, more severe legal punishment was associated with lower revenge feelings in victims of crime (5, 7a). However, one of the studies found that punishment severity did not significantly impact revenge feelings at a point several years after the trial (7b). The authors posited that harsher punishment could reduce revenge feelings in the short-term, but not in the long-term (7b). Additionally, in one of the studies (5), the relationship between punishment severity and revenge was no longer significant after controlling for PTSD symptoms. Indeed, there are many potential confounding factors here, which are not well-understood. For example, victims with a higher tendency to forgive may find that perpetrator punishment has minimal impact on revenge feelings (McCullough et al., 2001). There is a need for further research here, including confounding factors, to clarify the nature of the relationship between offender punishment and revenge.

Compensation and current juridical status were each examined in a single study. Higher satisfaction with compensation was linked to reduced revenge feelings in victims of crime at a point several years after the criminal trial (7b). This suggests that adequate financial compensation could reduce revenge feelings in crime victims. This accords with previous research on the goals of revenge in crime victims, which has found that victims often seek to redress the balance of gains and losses post-crime (Orth, 2004). Participants in the study may therefore have felt that their desire for revenge was satisfied, as the compensation they received redressed the balance of gains and losses. However, further research is needed to replicate this finding, since it was only examined in a single study. Additionally, one study found that the juridical status of the perpetrator was not related to victims’ revenge feelings. In other words, whether the offender had been found, convicted, or discharged did not impact victims’ revenge feelings (11). This is surprising, as one might expect victims to experience lower revenge desire where the offender has been convicted, since justice has already been done (Gerber & Jackson, 2013; Osgood, 2017). As with compensation, further research is needed to corroborate this finding.

A meta-analysis of four randomized controlled trials revealed that crime victims who experienced restorative justice conferences had lower desire for violent revenge than victims who had been through traditional criminal justice processes (9a, 9b, 9c, 9d). This indicates that restorative justice participation could reduce revenge desire in crime victims. The authors draw on cognitive behavioral therapy, to note that restorative justice could reduce the victim’s fear of the offender, thereby reducing their need to use revenge feelings as a coping mechanism post-crime (Rothbaum & Schwartz, 2002). This is based on the idea that restorative justice conferences facilitate exposure to the offender in a safe space. This enables victims to face their fears and de-conditions memories of the crime. Additionally, Sherman et al. (2005) posit that restorative justice could reduce victims’ self-blame for the crime. Victims often appear to blame themselves for the crime and feel ashamed that they were unable to defend themselves. During conferences, offenders can clarify that they did not target the victim for any particular reason. The offender can explain that they simply spotted an opportunity. Gaining this knowledge from restorative justice conferences could reduce victims’ feelings of weakness and their sense of shame around the crime, lowering revenge desire, as victims no longer need to rely on revenge fantasies to maintain their positive self-image (Bloom, 2001; Orth, 2004). Research by Funk et al. (2014) sheds further light on the mechanisms behind the link between restorative justice and reduced revenge desire. Funk et al. (2014) have found that enacting punishment can result in lowered desire for revenge when this punishment effectively communicates a moral message to the wrongdoer. If revenge desire is to be lowered, the offender must understand that their actions were wrong and signal to the victim that they will change their behavior in the future (Funk et al., 2014). Restorative justice conferences could therefore reduce revenge desire, as victims often receive an apology from the offender and offenders regularly commit to changing their behavior in the future (Suzuki & Jenkins, 2023). This meta-analysis of four randomized controlled trials indicates that restorative justice has the potential to reduce the desire for violent revenge in crime victims (9a, 9b, 9c, 9d).

### Demographic predictors

The majority of the included studies examined whether sociodemographic variables, including age and gender, predicted revenge desire in crime victims (1,3,4,5,7a,7b,10). Additionally, socio-economic status, past exposure to violence, and the influence of parents’ and friends’ aggression, were examined in a single study of adolescents (1). Not all of the included predictors were significant. See Table 2 and Table 4 for a summary of significant predictors.

Many of the included studies examined the influence of gender and age. For both age and gender, results were mixed. No clear pattern emerged. See Table 2 for more detail. This reflects the wider literature on revenge, in which the relationship between gender and revenge desire is disputed (Goldner et al., 2019). Previous studies have found that older age is linked to lower revenge desire (Cota-McKinley et al., 2001). However, no such effect was found in this review. Moreover, socio-economic status, measured using household income, was found to significantly impact revenge attitudes in a sample of adolescents who had been violently assaulted (1). Individuals from households with lower incomes were significantly more likely to support revenge. Boys with friends who engaged in aggressive behavior, and those young people who believed that their parents supported fighting, were also more likely to support revenge. This indicates that young people’s attitudes to revenge can be shaped by their perceptions of their parents’ support of fighting, as well as the aggression that they observe among their peers. Past exposure to violence was examined in the same study, and it did not have a significant impact on revenge attitudes. It should be noted, however, that these predictors were merely examined in a single study and further research is required to replicate the findings.

### Miscellaneous

Finally, one miscellaneous predictor, which fell outside of the categories, was identified (1). This was aggression. Trait aggression did not significantly impact attitudes to revenge in a sample of adolescents who had been violently assaulted. It was, however, significantly linked to stronger revenge attitudes in girls. This finding that trait aggression did not predict revenge attitudes in the overall sample contradicts previous literature, in which revenge and trait aggression have been found to be closely related (Chester & DeWall, 2017). However, whilst aggression was not a significant predictor, the authors noted a trend, whereby higher aggression scores were associated with higher approval of revenge.

### Limitations

The included studies had several limitations. The majority of studies were cross-sectional. Therefore, it is not possible to confirm causation. Additionally, whilst four randomized controlled trials demonstrated that restorative justice can reduce crime victims’ desire for violent revenge, these studies were not double-blinded. However, this deviation from the standard process was considered to be justified. Police and other staff within the criminal justice system needed to be aware of those participants who had been assigned to restorative justice conferences and those who had not, so that they could carry out their roles. Finally, several studies did not include confounding factors (1,2). Confounds are difficult to identify when it comes to revenge desire, so this was considered acceptable. As Barker et al. (2023) note, confounding factors are difficult to identify in areas where many attitudinal, behavioral and lifestyle factors may impact the variable of interest. As discussed above, revenge desire may be influenced by personality traits, political beliefs, cultural factors, sociodemographic factors, and life experiences (Jackson et al., 2019). Finally, the majority of the included studies (69.23%) used published questionnaires to assess participants’ revenge desire and attitudes. However, one paper, a meta-analysis of 4 randomized controlled trials, developed its own outcome measure, asking participants a single question about whether they wished they could physically harm or retaliate against the offender (9a, 9b, 9c, 9d). The authors’ use of a single outcome measure is a limitation. However, this was considered to be acceptable, as the question was clear, unidimensional and narrow in scope.

The current review also involved several limitations. Only studies published in English were included. This is a limitation because studies undertaken in other countries, or including cross-cultural analyses, may have been missed. The reviewed studies took place in a small number of countries. Most studies were undertaken in the United States of America, the Netherlands or Germany, and there were no cross-cultural analyses. Cross-cultural analyses could be important in this area. This is because attitudes to revenge seem to be shaped by culture. For example, in cultures of honor, retaliatory aggression is viewed more favorably (Cohen et al., 1996; Schumann & Ross, 2010). Vengeful behavior in punishment games also varies significantly across countries (Gächter & Herrmann, 2008). Moreover, the predictors of revenge could vary between cultures, with anger having been found to be a more significant motivator of revenge in individualistic cultures than in collectivist cultures (Shteynberg, 2005). Additionally, victims’ interactions with criminal justice systems vary significantly around the world (World Justice Project, 2019). They could therefore have different impacts on revenge feelings. As a result, country-specific and cross-cultural analyses are required here, and they are likely to have been missed by the current, English-language only review.

### Future research

Future research should focus on the relationship between mental health factors, and revenge desire and attitudes. There is a particular need to examine the role of anxiety and depression in revenge in adults, given that these were only examined in a single study of adolescents. As discussed above, the relationship between mental health and revenge desire is not well understood. There are limited studies examining it. And even where there are several studies on a particular mental health condition, for example on PTSD and revenge, the exact nature and direction of the relationship is unclear (Kunst, 2011). Yet understanding the relationship between revenge desire and mental health has the potential to improve support for the many victims of crime who report struggling with their mental health (Kilpatrick & Acierno, 2003). This knowledge could inform both clinicians and victim support services.

Additionally, further research is needed into the impact that the criminal justice system could be having on crime victims’ revenge desire. Several studies in the current review examined limited aspects of this, but it is clear that the relationship between victims’ experiences within the criminal justice system and revenge are not well-understood (Orth, 2004). It is also likely to vary across countries, in which victims can have significantly different experiences of navigating justice systems (World Justice Project, 2019). Research could be expanded to include more justice-related predictors, such as victims’ subjective levels of satisfaction with their experiences of the criminal justice system, or the role of victim impact statements. Research by Gollwitzer et al. (2011) and Funk et al. (2014) has found that delivering a moral message to the wrongdoer is an important part of what makes revenge satisfying. This suggests that victim impact statements could potentially reduce revenge feelings in crime victims.

### Practical applications

This review has highlighted the lack of research on the predictors of revenge desire and attitudes in victims of crime. It is therefore difficult to make practical recommendations, as further research is required to expand on many of the identified predictors. However, several tentative recommendations can be made. Practitioners and support services should bear in mind that victims of crime may be at greater risk of experiencing long-term revenge desire than the general population, and that revenge desire is associated with poor mental health outcomes, in particular PTSD. As a result, victims of crime may need support with revenge feelings. This could come in the form of early intervention, or long-term therapeutic support. Revenge feelings are stigmatized, which could prevent victims from seeking support (Gollwitzer, 2009). Breaking down the stigma associated with revenge desire is therefore vital. In addition, interactions with the criminal justice system impact victims’ revenge desire. In recent years, national governments, including that of the United Kingdom, have implemented programs of reform which have aimed to improve the criminal justice experience for victims of crime (Baird, 2022). Yet these programs have failed to consider how the criminal justice system could be impacting victims’ and survivors’ revenge desire and attitudes. The current review highlights that policies which seek to improve crime victims’ experiences of the criminal justice system should consider how the criminal justice system can impact victims’ revenge desire. For example. participating in restorative justice was found to lower victims’ desire for violent revenge (9a, 9b, 9c, 9d). The benefits of restorative justice are widely cited. For example, reducing victims’ fear of the offender, lowering recidivism, and helping offenders to recognize the consequences of their actions (Crown Prosecution Service, 2023). However, this review highlights an underrecognized benefit of restorative justice: reducing victims’ revenge desire. It indicates that access to restorative justice should be expanded. This is because it could have benefits for victims’ mental health, by reducing revenge desire. And given that criminal victimization is linked to an increased risk of revenge-motivated violent offending by victims, it could also reduce violent offending. Based on existing literature, it is difficult to paint a comprehensive picture of those victims who are at the greatest risk of experiencing revenge desire or having attitudes supportive of revenge. Yet such a picture could inform violence prevention interventions and help to target them effectively. These interventions could take the form of community activities, or even addiction-treatment techniques (Chester & DeWall, 2017; D. B. Lee et al., 2022).

## Conclusion

Whilst there have been some promising findings, for example the finding that restorative justice participation could reduce crime victims’ desire for violent revenge (9a, 9b, 9c, 9d), the current review has highlighted the lack of quantitative research on revenge desire and attitudes in victims of crime. Several categories of predictors were identified, namely 1) offence-related predictors, 2) mental health predictors, 3) criminal justice system predictors, and 4) demographic predictors. These categories can be used to guide future research on the predictors of revenge desire in crime victims. There is a pressing need for further research here, particularly on mental health predictors. Research on the relationship between mental health and revenge feelings could improve support for victims of crime, who frequently report struggling with their mental health (Kilpatrick & Acierno, 2003). Given the fact that revenge is a significant motivator of violence, this research could also reduce instances of violence by informing violence intervention strategies (Jackson et al., 2019; Lee et al., 2022).

## Data Availability

Data sharing not applicable to this article as no datasets were generated or analyzed during the current study.

## Appendix A

### Searches Conducted

#### APA PsycInfo <1967 to February Week 2 2024>

APA PsycInfo was searched in February 2024, obtaining 558 results

1 ((revenge or vengeance or retaliate or retaliation or vengefulness) and victim* and (homicide or murder or manslaughter or infanticide or filicide or dangerous driving or inconsiderate driving or injury or assault or harassment or hate crime or stalking or sexual assault or sex* offence or sex* offense or sexual abuse or rape or grooming or exposure or voyeurism or violent crime or violence or burglary or robbery or theft or fraud or kidnapping or blackmail or vandalism or arson or firearms or weapon* or criminal damage or child abuse or cybercrime or domestic violence or terrorism or physical abuse or kidnapping or incest or human trafficking or antisocial behaviour or antisocial behavior or criminal behaviour or criminal behavior or crime)).mp. [mp=title, abstract, heading word, table of contents, key concepts, original title, tests & measures, mesh word] 558

#### APA PsycArticles Full Text

APA PsycArticles was searched in February 2024, obtaining 1362 results

1 ((revenge or vengeance or retaliate or retaliation or vengefulness) and victim* and (homicide or murder or manslaughter or infanticide or filicide or dangerous driving or inconsiderate driving or injury or assault or harassment or hate crime or stalking or sexual assault or sex* offence or sex* offense or sexual abuse or rape or grooming or exposure or voyeurism or violent crime or violence or burglary or robbery or theft or fraud or kidnapping or blackmail or vandalism or arson or firearms or weapon* or criminal damage or child abuse or cybercrime or domestic violence or terrorism or physical abuse or kidnapping or incest or human trafficking or antisocial behaviour or antisocial behavior or criminal behaviour or criminal behavior or crime)).mp. [mp=title, abstract, full text, caption text] 1362

#### Ovid MEDLINE(R) ALL <1946 to February 8, 2024>

MEDLINE was searched in February 2024, obtaining 253 results

1 ((revenge or vengeance or retaliate or retaliation or vengefulness) and victim* and (homicide or murder or manslaughter or infanticide or filicide or dangerous driving or inconsiderate driving or injury or assault or harassment or hate crime or stalking or sexual assault or sex* offence or sex* offense or sexual abuse or rape or grooming or exposure or voyeurism or violent crime or violence or burglary or robbery or theft or fraud or kidnapping or blackmail or vandalism or arson or firearms or weapon* or criminal damage or child abuse or cybercrime or domestic violence or terrorism or physical abuse or kidnapping or incest or human trafficking or antisocial behaviour or antisocial behavior or criminal behaviour or criminal behavior or crime)).mp. [mp=title, book title, abstract, original title, name of substance word, subject heading word, floating sub-heading word, keyword heading word, organism supplementary concept word, protocol supplementary concept word, rare disease supplementary concept word, unique identifier, synonyms, population supplementary concept word, anatomy supplementary concept word] 253

#### Web of Science

Web of Science was searched on 31st January 2024, obtaining 723 results

# Web of Science Search Strategy (v0.1)
# Database: Web of Science Core Collection
# Entitlements:
  - WOS.SCI: 1970 to 2024
  - WOS.AHCI: 1975 to 2024
  - WOS.ESCI: 2015 to 2024
  - WOS.ISTP: 1990 to 2024
  - WOS.SSCI: 1970 to 2024
  - WOS.ISSHP: 1990 to 2024
  # Searches:

Search: (revenge OR vengeance OR vengefulness OR retaliat*) AND victim* AND (homicide OR murder OR manslaughter OR infanticide OR filicide OR “dangerous driving” OR “inconsiderate driving” OR injury OR assault OR harassment OR “hate crime” OR stalk* OR “sexual assault” OR “sex* offence” OR “sexual abuse” OR rape OR groom* OR exposure OR voyeur* OR “violent crime” OR violence OR burglary OR robbery OR theft OR fraud OR blackmail OR vandal* OR arson OR firearm* OR weapon* OR “criminal damage” OR “child abuse” OR cybercrime OR “domestic violence” OR terror* OR “physical abuse” OR kidnap* OR incest OR traffick* OR “antisocial behavio*” OR “criminal behavio*” OR crime) (All Fields) Date Run: Wed Jan 31 2024 15:13:53 GMT+0000 (Greenwich Mean Time) Results: 723

#### SCOPUS

SCOPUS was searched in February 2024, obtaining 793 results

(ALL (revenge OR vengeance OR vengefulness OR retaliate OR retaliation) AND TITLE-ABS-KEY (victim*) AND TITLE-ABS-KEY (homicide OR murder OR manslaughter OR infanticide OR filicide OR “dangerous driving” OR “inconsiderate driving” OR injury OR assault OR harassment OR hate AND crime OR stalk* OR “sexual assault” OR “sex* offence” OR “sex* offense” OR “sexual abuse” OR rape OR groom* OR exposure OR voyeur OR “violent crime” OR violence OR burglary OR robbery OR theft OR fraud OR blackmail OR vandal* OR arson OR firearm* OR weapon* OR “criminal damage” OR “child abuse” OR cybercrime OR “domestic violence” OR terror OR “physical abuse” OR kidnap* OR incest OR traffick* OR “antisocial behavio*” OR “criminal behavio*” OR crime)

## References

[bibr1-00332941241313032] Achenbach T. M. (1991). Manual for the child behaviour check-list/4-18 and 1991 profile. Burlington, VT: University of Vermont, Department of Psychiatry.

[bibr2-00332941241313032] Ames D. L. Fiske S. T. (2013). Intentional harms are worse, even when they’re not. Psychological Science, 24(9), 9. 10.1177/0956797613480507 PMC4470288 23878021

[bibr3-00332941241313032] Appelo M. (2005). Positieve Uitkomsten Lijst (PUL): Handleiding.

[bibr4-00332941241313032] Appelo M. Harkema-Schouten P. (2003). De positieve Uitkomsten Lijst (PUL). Gedragstherapie.

[bibr5-00332941241313032] Aquino K. Tripp T. M. Bies R. J. (2006). Getting even or moving on? Power, procedural justice, and types of offense as predictors of revenge, forgiveness, reconciliation, and avoidance in organizations. Journal of Applied Psychology, 91(3), 653–668. 10.1037/0021-9010.91.3.653 16737361

[bibr6-00332941241313032] Arntz A. (1993). Dutch translation of the PSS-SR. Universiteit Maastricht.

[bibr7-00332941241313032] Baird V. (2022). 2021/22 annual report of the victims’ commissioner for england and wales. https://cloud-platform-e218f50a4812967ba1215eaecede923f.s3.amazonaws.com/uploads/sites/6/2022/06/MOJ7216_Victims-commisioner-Annual-report_AW-WEB.pdf

[bibr8-00332941241313032] Barcaccia B. Salvati M. Pallini S. Saliani A. M. Baiocco R. Vecchio G. M. (2022). The bitter taste of revenge: Negative affect, depression and anxiety. Current Psychology, 41(3), 3. 10.1007/s12144-020-00643-1

[bibr9-00332941241313032] Barker T. H. Stone J. C. Sears K. Klugar M. Tufanaru C. Leonardi-Bee J. Aromataris E. Munn Z. (2023). The revised JBI critical appraisal tool for the assessment of risk of bias for randomized controlled trials. JBI Evidence Synthesis, 21(3), 3. 10.11124/JBIES-22-00430 36727247

[bibr10-00332941241313032] Berry J. W. Worthington E. L. O’Connor L. E. Parrott L. Wade N. G. (2005). Forgivingness, vengeful rumination, and affective traits. Journal of Personality, 73(1), 1. 10.1111/j.1467-6494.2004.00308.x 15660677

[bibr11-00332941241313032] Blevins C. A. Weathers F. W. Davis M. T. Witte T. K. Domino J. L. (2015). The posttraumatic stress disorder checklist for *DSM‐5* (PCL‐5): Development and initial psychometric evaluation. Journal of Traumatic Stress, 28(6), 489–498. 10.1002/jts.22059 26606250

[bibr12-00332941241313032] Bloom S. L. (2001). Commentary. Journal of Emotional Abuse, 2(4), 4. 10.1300/J135v02n04_06

[bibr13-00332941241313032] Boon S. D. Deveau V. L. Alibhai A. M. (2009). Payback: The parameters of revenge in romantic relationships. Journal of Social and Personal Relationships, 26(6–7), 747–768. 10.1177/0265407509347926

[bibr14-00332941241313032] Boon S. D. Yoshimura S. M. (2020). Revenge as social interaction: Merging social psychological and interpersonal communication approaches to the study of vengeful behavior. Social and Personality Psychology Compass, 14(9), 9. 10.1111/spc3.12554

[bibr15-00332941241313032] Carmody P. Gordon K. (2011). Offender variables: Unique predictors of benevolence, avoidance, and revenge? Personality and Individual Differences, 50(7), 1012–1017. 10.1016/j.paid.2010.12.037

[bibr16-00332941241313032] Chester D. S. DeWall C. N. (2017). Combating the sting of rejection with the pleasure of revenge: A new look at how emotion shapes aggression. Journal of Personality and Social Psychology, 112(3), 413–430. 10.1037/pspi0000080 27808535

[bibr17-00332941241313032] Cohen D. Nisbett R. E. Bowdle B. F. Schwarz N. (1996). Insult, aggression, and the southern culture of honor: An ‘experimental ethnography. Journal of Personality and Social Psychology, 70(5), 945–960. 10.1037/0022-3514.70.5.945 8656339

[bibr18-00332941241313032] Copeland-Linder N. Jones V. C. Haynie D. L. Simons-Morton B. G. Wright J. L. Cheng T. L. (2007). Factors associated with retaliatory attitudes among African American adolescents who have been assaulted. Journal of Pediatric Psychology, 32(7), 7. 10.1093/jpepsy/jsm007 17403911

[bibr19-00332941241313032] Cota-McKinley A. L. Woody W. D. Bell P. A. (2001). Vengeance: Effects of gender, age, and religious background. Aggressive Behavior, 27(5), 5. 10.1002/ab.1019

[bibr20-00332941241313032] Crombag H. Rassin E. Horselenberg R. (2003). On vengeance. Psychology, Crime & Law, 9(4), 4. 10.1080/1068316031000068647

[bibr21-00332941241313032] Crown Prosecution Service . (2023). Restorative justice. https://cps.gov.uk/legal-guidance/restorative-justice

[bibr82-00332941241313032] Davidson M. Lozano N. M. Cole B. P. Gervais S. J. , . (2013). Associations between women’s experiences of sexual violence and forgiveness. Violence and Victims, 28(6), 1041–1053. 10.1891/0886-6708.vv-d-12-00075 24547679

[bibr22-00332941241313032] DiBlasi T. Kassinove H. (2022). An exploratory analysis of components of revenge experiences. Journal of Aggression, Maltreatment & Trauma, 31(7), 873–887. 10.1080/10926771.2021.2013376

[bibr23-00332941241313032] DiBlasi T. Wydo M. Smith K. (2024). An exploratory analysis of vengeful episodes in prisoners. Journal of Rational-Emotive & Cognitive-Behavior Therapy, 42(4), 930–944. 10.1007/s10942-024-00555-0

[bibr89-00332941241313032] Ehlers A. Hackmann A. Michael T. , . (2004). Intrusive re‐experiencing in post‐traumatic stress disorder: Phenomenology, theory, and therapy. Memory, 12(4), 403–415. 10.1080/09658210444000025 15487537

[bibr24-00332941241313032] Erikson E. H. (1968). Identity: Youth and crisis. Norton & Co.

[bibr25-00332941241313032] Evans C. Ehlers A. Mezey G. Clark D. M. (2007). Intrusive memories and ruminations related to violent crime among young offenders: Phenomenological characteristics. Journal of Traumatic Stress, 20(2), 2. 10.1002/jts.20204 17427909

[bibr26-00332941241313032] Feuer A. Singer J. (2017). A Brooklyn murder’s decades-old origins in rural China. The New York Times. https://www.nytimes.com/2017/07/16/nyregion/a-brooklyn-murders-decades-old-origins-in-rural-china.html

[bibr27-00332941241313032] Field C. (2012). An examination of forgiveness and revenge in victims of crime.

[bibr28-00332941241313032] Finkel E. J. Rusbult C. E. Kumashiro M. Hannon P. A. (2002). Dealing with betrayal in close relationships: Does commitment promote forgiveness? Journal of Personality and Social Psychology, 82(6), 6. 10.1037//0022-3514.82.6.956 12051583

[bibr29-00332941241313032] Foa E. B. Riggs D. S. Dancu C. V. Rothbaum B. O. (1993). Reliability and validity of a brief instrument for assessing post-traumatic stress disorder. Journal of Traumatic Stress, 6(4), 459–473.

[bibr83-00332941241313032] Franczyk-Glita J. Müldner-Nieckowski L. Śmierciak N. Daren A. Furgał M. , . (2018). Psychoterapia, 184, 65–78.

[bibr30-00332941241313032] Funk F. McGeer V. Gollwitzer M. (2014). Get the message: Punishment is satisfying if the transgressor responds to its communicative intent. Personality and Social Psychology Bulletin, 40(8), 986–997. 10.1177/0146167214533130 24789809

[bibr31-00332941241313032] Gächter S. Herrmann B. (2008). Reciprocity, culture and human cooperation: Previous insights and a new cross-cultural experiment. Philosophical Transactions of the Royal Society B: Biological Sciences, 364(1518), 1518. 10.1098/rstb.2008.0275 PMC2689715 19073476

[bibr87-00332941241313032] Gaś Z. (1988). Inwentarz psychologiczny Syndromu Agresji [The Psychological Inventory of Aggression Syndrome]. Przegląd Psychologiczny, 23, 143–154.

[bibr32-00332941241313032] Gerber M. M. Jackson J. (2013). Retribution as revenge and retribution as just deserts. Social Justice Research, 26(1), 61–80. 10.1007/s11211-012-0174-7

[bibr84-00332941241313032] Gerlsma C. Lugtmeyer V. (2016). Offense type as determinant of revenge and Forgiveness After Victimization: Adolescents’ responses to injustice and aggression. Journal of School Violence, 17(1), 16–27. 10.1080/15388220.2016.1193741

[bibr33-00332941241313032] Gerlsma C. Lugtmeyer V. (2018). Offense type as determinant of revenge and forgiveness after victimization: Adolescents’ responses to injustice and aggression. Journal of School Violence, 17(1), 1. 10.1080/15388220.2016.1193741

[bibr34-00332941241313032] Goldner L. Lev-Wiesel R. Simon G. (2019). Revenge fantasies after experiencing traumatic events: Sex differences. Frontiers in Psychology, 10, 886. 10.3389/fpsyg.2019.00886 PMC6520653 31143138

[bibr35-00332941241313032] Gollwitzer M. (2009). Justice and revenge. In Oswald M. E. Bieneck S. Hupfeld-Heinemann J. (Eds.), Social psychology of punishment of crime (p. 137). John Wiley & Sons.

[bibr36-00332941241313032] Gollwitzer M. Meder M. Schmitt M. (2011). What gives victims satisfaction when they seek revenge? European Journal of Social Psychology, 41(3), 3. 10.1002/ejsp.782

[bibr37-00332941241313032] Gray K. Wegner D. M. (2009). Moral typecasting: Divergent perceptions of moral agents and moral patients. Journal of Personality and Social Psychology, 96, 505–520. 10.1037/a0013748 19254100

[bibr38-00332941241313032] Hannon L. (2002). Criminal opportunity theory and the relationship between poverty and property crime. Sociological Spectrum, 22(3), 363–381. 10.1080/02732170290062676

[bibr39-00332941241313032] Heber A. (2014). Good versus bad? Victims, offenders and victim-offenders in Swedish crime policy. European Journal of Criminology, 11(4), 410–428. 10.1177/1477370813503920

[bibr40-00332941241313032] Horowitz M. J. (2007). Understanding and ameliorating revenge fantasies in psychotherapy. American Journal of Psychiatry, 164(1), 1. 10.1176/ajp.2007.164.1.24 17202539

[bibr41-00332941241313032] Hruschka D. J. Henrich J. (2006). Friendship, cliquishness, and the emergence of cooperation. Journal of Theoretical Biology, 239(1), 1. 10.1016/j.jtbi.2005.07.006 16102781

[bibr42-00332941241313032] Jackson J. C. Choi V. K. Gelfand M. J. (2019). Revenge: A multilevel review and synthesis. Annual Review of Psychology, 70(1), 1. 10.1146/annurev-psych-010418-103305 30609911

[bibr43-00332941241313032] Kilpatrick D. G. Acierno R. (2003). Mental health needs of crime victims: Epidemiology and outcomes. Journal of Traumatic Stress, 16(2), 2. 10.1023/A:1022891005388 12699200

[bibr44-00332941241313032] Koss M. P. Abbey A. Campbell R. Cook S. Norris J. Testa M. Ullman S. West C. White J. (2007). Revising the ses: A collaborative process to improve assessment of sexual aggression and victimization. Psychology of Women Quarterly, 31(4), 357–370. 10.1111/j.1471-6402.2007.00385.x

[bibr45-00332941241313032] Kunst M. J. J. (2011). PTSD symptom clusters, feelings of revenge, and perceptions of perpetrator punishment severity in victims of interpersonal violence. International Journal of Law and Psychiatry, 34(5), 5. 10.1016/j.ijlp.2011.08.003 21903272

[bibr46-00332941241313032] Lee D. B. Schmidt C. J. Heinze J. E. Carter P. M. Cunningham R. M. Walton M. A. Zimmerman M. A. (2022). Retaliatory attitudes as mediator of exposure to violence and firearm aggression among youth: The protective role of organized activity involvement. Developmental Psychology, 58(5), 990–1002. 10.1037/dev0001339 PMC9716642 35377700

[bibr47-00332941241313032] Lee T. L. Gelfand M. J. Kashima Y. (2014). The serial reproduction of conflict: Third parties escalate conflict through communication biases. Journal of Experimental Social Psychology, 54, 68–72. 10.1016/j.jesp.2014.04.006

[bibr85-00332941241313032] Lenferink L. I. M Nickerson A. Kashyap S. De Keijser J. Boelen P. A. , . (2022). Associations of dimensions of anger with distress following traumatic bereavement. Psychological Trauma Theory Research Practice and Policy, 16(2), 176–183. 10.1037/tra0001275 35511540

[bibr48-00332941241313032] Lopes Cardozo B. Vergara A. Agani F. Gotway C. A. (2000). Mental health, social functioning, and attitudes of Kosovar Albanians following the war in Kosovo. JAMA, 284(5), 5. 10.1001/jama.284.5.569 10918702

[bibr49-00332941241313032] Maercker A. Schützwohl M. (1998). Erfassung von psychischen Belastungsfolgen: Die Impact of Event Skala-revidierte Version (IES-R). [Assessment of post-traumatic stress reactions: The Impact of Event Scale-Revised (IES-R).]. Diagnostica, 44(3), 130–141.

[bibr50-00332941241313032] McCullough M. E. Bellah C. G. Kilpatrick S. D. Johnson J. L. (2001). Vengefulness: Relationships with forgiveness, rumination, well-being, and the big five. Personality and Social Psychology Bulletin, 27(5), 5. 10.1177/0146167201275008

[bibr86-00332941241313032] McCullough M. E. Rachal K. C. Sandage S. J. Worthington E. L. Brown S. W. Hight T. L. (1998). Transgression-Related interpersonal motivations inventory. PsycTESTS Dataset. 10.1037/t03960-000 9914668

[bibr51-00332941241313032] Michael T. Halligan S. L. Clark D. M. Ehlers A. (2007). Rumination in posttraumatic stress disorder. Depression and Anxiety, 24(5), 5. 10.1002/da.20228 17041914

[bibr52-00332941241313032] Moola S. Munn Z. Tufanaru C. Aromataris E. Sears K. Sfetcu R. Currie M. Qureshi R. Mattis P. Lisy K. Mu P. F. (2020). Chapter 7: Systematic reviews of etiology and risk. Aromataris E. Munn Z. (Eds.), JBI Manual for Evidence Synthesis.

[bibr53-00332941241313032] Munn Z. Peters M. D. J. Stern C. Tufanaru C. McArthur A. Aromataris E. (2018). Systematic review or scoping review? Guidance for authors when choosing between a systematic or scoping review approach. BMC Medical Research Methodology, 18(1), 143. 10.1186/s12874-018-0611-x PMC6245623 30453902

[bibr54-00332941241313032] New York Police Department . (2012). Murder in New York city. New York Police Department. https://www.nyc.gov/site/nypd/stats/reports-analysis/archive.page

[bibr55-00332941241313032] Orpinas P. Murray N. Kelder S. (1999). Parental influences on students’ aggressive behaviors and weapon carrying. Health Education & Behavior, 26(6), 774–787. 10.1177/109019819902600603 10608570

[bibr56-00332941241313032] Orpinas P. K. (1993). Skills training and social influences for violence prevention in middle schools: A curriculum evaluation. The University of Texas School of Public Health.

[bibr57-00332941241313032] Orth U. (2003). Punishment goals of crime victims. Law and Human Behavior, 27(2), 2. 10.1023/A:1022547213760 12733420

[bibr58-00332941241313032] Orth U. (2004). Does perpetrator punishment satisfy victims’ feelings of revenge? Aggressive Behavior, 30(1), 1. 10.1002/ab.20003

[bibr88-00332941241313032] Orth U. Maercker A. (2009). Posttraumatic anger in crime victims: Directed at the perpetrator and at the self. Journal of Traumatic Stress, 22(2), 158–161. 10.1002/jts.20392 19283835

[bibr59-00332941241313032] Orth U. Montada L. Maercker A. (2006). Feelings of revenge, retaliation motive, and posttraumatic stress reactions in crime victims. Journal of Interpersonal Violence, 21(2), 2. 10.1177/0886260505282286 16368763

[bibr60-00332941241313032] Osgood J. M. (2017). Is revenge about retributive justice, deterring harm, or both? Social and Personality Psychology Compass, 11(1), e12296. 10.1111/spc3.12296

[bibr61-00332941241313032] Ouzzani M. Hammady H. Fedorowicz Z. Elmagarmid A. (2016). Rayyan—a web and mobile app for systematic reviews. 10.1186/s13643-016-0384-4 PMC5139140 27919275

[bibr62-00332941241313032] Parsons J. Bergin T. (2010). The impact of criminal justice involvement on victims’ mental health. Journal of Traumatic Stress, 23(2), 182–188. 10.1002/jts.20505 20225265

[bibr63-00332941241313032] Prigerson H. G. Maciejewski P. K. Reynolds C. F. Bierhals A. J. Newsom J. T. Fasiczka A. Frank E. Doman J. Miller M. (1995). Inventory of complicated grief: A scale to measure maladaptive symptoms of loss. Psychiatry Research, 59(1–2), 65–79. 10.1016/0165-1781(95)02757-2 8771222

[bibr64-00332941241313032] Rasmussen K. (2016). Entitled vengeance: A meta-analysis relating narcissism to provoked aggression. Aggressive Behavior, 42(4), 362–379. 10.1002/ab.21632 26522921

[bibr65-00332941241313032] Richters J. E. Martinez P. (1993). The nimh community violence Project: I. Children as victims of and witnesses to violence. Psychiatry, 56(1), 7–21. 10.1080/00332747.1993.11024617 8488215

[bibr66-00332941241313032] Roseman I. J. Wiest C. Swartz T. S. (1994). Phenomenology, behaviors, and goals differentiate discrete emotions. Journal of Personality and Social Psychology, 67, 206–221. 10.1037/0022-3514.67.2.206

[bibr67-00332941241313032] Rothbaum B. O. Schwartz A. C. (2002). Exposure therapy for posttraumatic stress disorder. American Journal of Psychotherapy, 56(1), 1. 10.1176/appi.psychotherapy.2002.56.1.59 11977784

[bibr68-00332941241313032] Schumann K. Ross M. (2010). The benefits, costs, and paradox of revenge. Social and Personality Psychology Compass, 4(12), 12. 10.1111/j.1751-9004.2010.00322.x

[bibr69-00332941241313032] Sherman L. W. Strang H. Angel C. Woods D. Barnes G. C. Bennett S. Inkpen N. (2005). Effects of face-to-face restorative justice on victims of crime in four randomized, controlled trials. Journal of Experimental Criminology, 1(3), 3. 10.1007/s11292-005-8126-y

[bibr70-00332941241313032] Shteynberg G. (2005). The cultural psychology of revenge in the Unites [ie United] States and South Korea.

[bibr71-00332941241313032] Simons-Morton B. Crump A. D. Haynie D. L. Saylor K. E. Eitel P. Yu K. (1999). Psychosocial, school, and parent factors associated with recent smoking among early-adolescent boys and girls. Preventive Medicine, 28(2), 138–148. 10.1006/pmed.1998.0404 10048105

[bibr72-00332941241313032] Stuckless N. (1997). The influence of anger, perceived injustice, revenge, and time on the quality of life of survivor-victims. https://library-archives.canada.ca/eng/services/services-libraries/theses/Pages/item.aspx?idNumber=46560029

[bibr73-00332941241313032] Stuckless N. (1997). The influence of anger, perceived injustice, revenge, and time on the quality of life of survivor-victims. https://library-archives.canada.ca/eng/services/services-libraries/theses/Pages/item.aspx?idNumber=46560029

[bibr74-00332941241313032] Stuckless N. Goranson R. (1994). A selected bibliography of literature on revenge. Psychological Reports, 75(2), 2. 10.2466/pr0.1994.75.2.803 7862788

[bibr75-00332941241313032] Suzuki M. Jenkins T. (2023). Apology–forgiveness cycle in restorative justice, but how? International Review of Victimology, 29(2), 259–276. 10.1177/02697580221079994

[bibr76-00332941241313032] Thomaes S. (2006). Narcissism, shame, and aggression in early adolescence: On vulnerable children.

[bibr77-00332941241313032] Tripp T. M. Bies R. J. (2010). “Righteous” anger and revenge in the workplace: The fantasies, the feuds, the forgiveness. In Potegal M. Stemmler G. Spielberger C. (Eds.), International handbook of anger (pp. 413–431): Springer. 10.1007/978-0-387-89676-2_24

[bibr78-00332941241313032] van Denderen M. de Keijser J. Gerlsma C. Huisman M. Boelen P. A. (2014). Revenge and psychological adjustment after homicidal loss. Aggressive Behavior, 40(6), 6. 10.1002/ab.21543 24910007

[bibr79-00332941241313032] Vossekuil B. Fein R. A. Reddy M. Borum R. Modzeleski W. (2004). The final report and findings of the safe school initiative: Implications for the prevention of school attacks in the United States. United States Secret Service and United States Department of Education.

[bibr80-00332941241313032] Weiss D. S. Marmar C. R. (1997). The impact of event scale—revised. In Assessing psychological trauma and PTSD (pp. 399–411). The Guilford Press.

[bibr81-00332941241313032] World Justice Project . (2019, February 13). What the data says about criminal justice systems around the world. World Justice Project. https://worldjusticeproject.org/news/what-data-says-about-criminal-justice-systems-around-world

